# A physiological sign that mimics lung point in critical care ultrasonography

**DOI:** 10.1186/s13054-015-0863-3

**Published:** 2015-03-30

**Authors:** Zhongheng Zhang, Lin Chen

**Affiliations:** Department of Critical Care Medicine, Jinhua Municipal Central Hospital, Jinhua Hospital of Zhejiang University, 351#, Mingyue street, 321000 Zhejiang, P.R. China

Ultrasound has been widely used in the critical care setting for timely and accurate diagnosis of life-threatening conditions. Pneumothorax is one such condition and typically is confirmed by the presence of the following ultrasonographic findings: abolished lung sliding or lung pulsing, a stratosphere sign, the absence of B lines, and the presence of lung point [1,2]. In particular, lung point is demonstrated to have 100% specificity for the diagnosis of pneumothorax [3]. Here, we present a sign that mimicked the findings of lung point but that was identified in healthy lung. To the best of our knowledge [4,5], this has never been described.

The study was approved by the ethics committee of Jinhua Municipal Central Hospital, and informed consent to publish was obtained from the patient. Lung ultrasonography was performed in a 79-year-old male patient by using a vascular probe (M-Turbo, SonoSite, Bothell, WA, USA). The left lung was scanned longitudinally, and lung point was identified at the 4th and 5th intercostal space in the middle clavicle line. Real-time mode showed intermittent eradication of cardiac pulse by the moving lung with respiration cycle (Figure 1a). The sand-like appearance of the field below the pleural line indicated normal aerated pulmonary tissue. A video clip showed cyclic movement of the lung edge with respiration (Addititonal file 1). The pleural line with lung sliding can be visualized on the left screen, and there is no lung sliding to the right of the lung edge. Subsequent computed tomography ruled out pneumothorax (Figure 1b).

**Figure 1:**
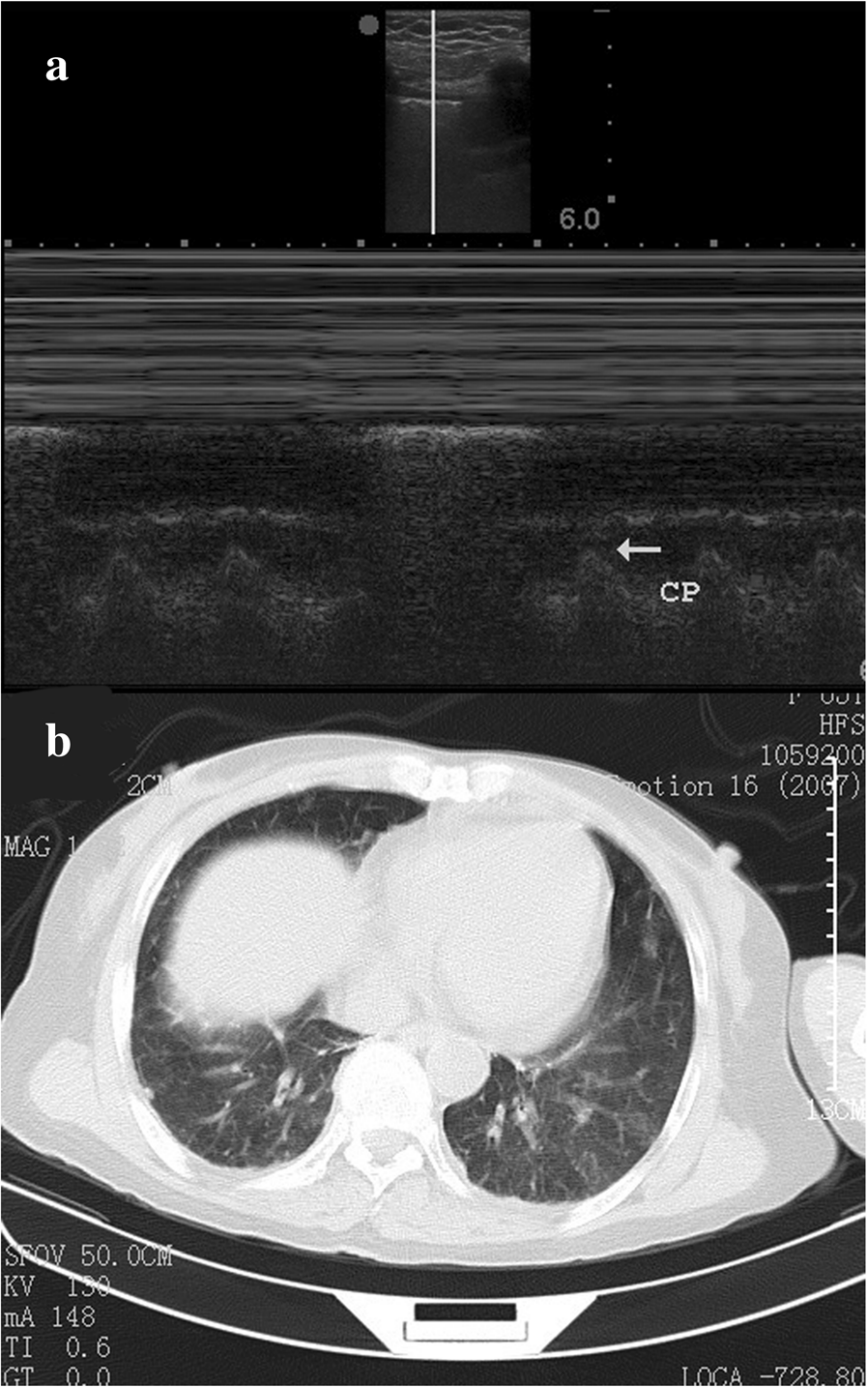
**Real-time mode ultrasonography and computed tomography. (a)** Real-time mode showed intermittent eradication of cardiac pulse (CP) by the moving lung with respiration cycle. **(b)** Subsequent computed tomography ruled out pneumothorax.

The physiological sign found in our report is thought to be formed at the mediastinal pleura, where visceral pleura have contact with soft tissue of the mediastinum. The lung expanded cyclically with inspiration, creating the appearance of a lung point sign on the ultrasound. The physiological sign differs from pneumothorax lung point in that soft tissue with cardiac pulse can be visualized at the no-lung region whereas in pneumothorax the no-lung region typically shows an A-line pattern without lung sliding. A lung point is seen at the transition of a lung image (B lines, consolidation, or sliding + A lines) with an image suggestive of pneumothorax (absence of lung sliding + A lines) [6]. The video does not show the absence of lung sliding + A lines. Instead, where there should be A lines (if this were a lung point), the video shows cardiac motion. Clinicians should be cautious in making a diagnosis of pneumothorax when they see this physiological sign.

## Additional file

Additional file 1:
**Physiological sign that mimics lung point.** The pleural line with lung sliding can be visualized on the left screen and there is no lung sliding to the right of the lung edge.
